# Supplementary material for: Ln3+-Induced Diblock Copolymeric Aggregates for Fully Flexible Tunable White-Light Materials
Source: Nanomaterials (Basel). 2019 Mar 5;9(3):363. doi: 10.3390/nano9030363 (PMC6474134; doi:10.3390/nano9030363)
Supplement: Supplementary file 1 [file nanomaterials-09-00363-s001.pdf]

Electronic Supplementary Information (ESI) for:
Ln^{3+}-Induced Diblock Copolymeric Aggregates for Full Flexible Tunable White-Light Materials

Xinzhi Wang,[a] Jianguo Tang,[*a] Guanghui Wang,[a] Wei Wang,[a] Junjie Ren,[a] Yao Wang,[a] Wenfei Shen,[a] Linjun Huang,[a] and Laurence A. Belfiore[*a,b]

Table of Contents

Figure S1. 1H NMR spectra of (a) 4-cyano-4-ethyl trithiopentanoic acid and (b) RAFT agent.
Figure S2. EDS spectrum of BCPs-Ln3+-Phen (Eu:Tb=1:1).
Table S1. Molar ratios of Eu3+, Tb3+, "phen", PS-b-PAA and their concentrations in DMF solutions before reaction.

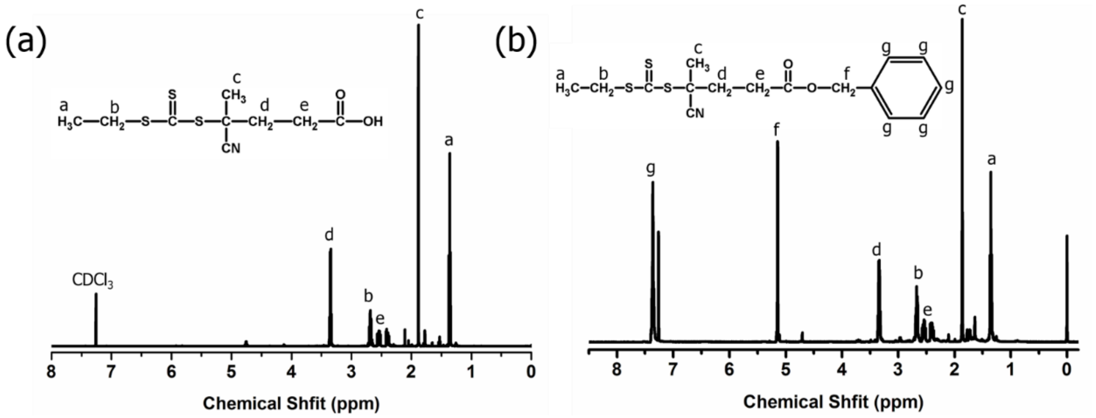

Figure 1. ^{1}H NMR spectra of (a) 4-cyano-4-ethyl trithiopentanoic acid and (b) RAFT agent.

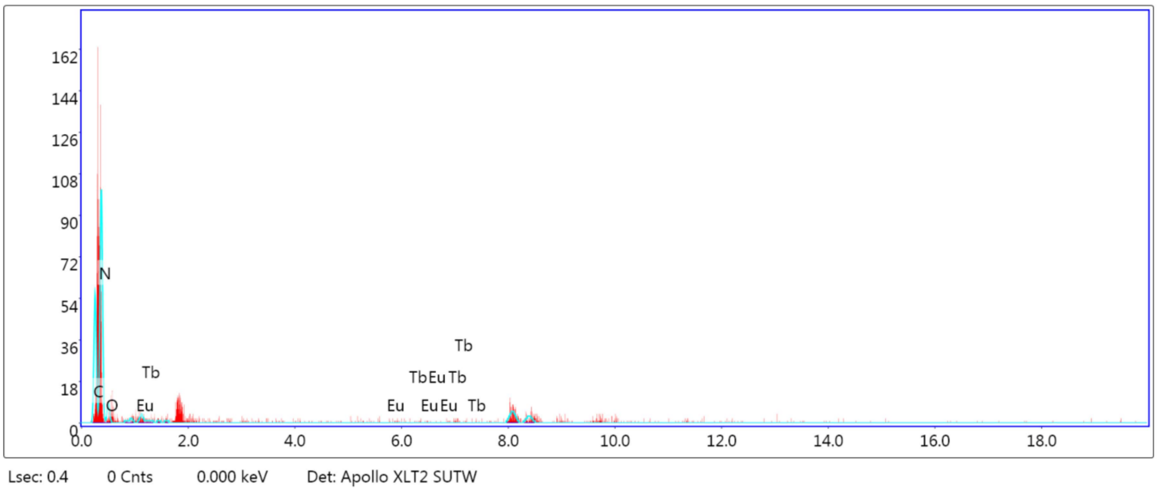

Figure S2. EDS spectrum of BCPs-Ln3+-Phen (Eu:Tb=1:1)

Table S1. Molar ratios of Eu3+, Tb3+, "phen", PS-b-PAA and their concentrations in DMF solutions before reaction.

Sample	$C_{PS-b-PAA}$ (mol/L)	C_{Eu} (mol/L)	C_{Tb} (mol/L)	C_{Phen} (mol/L)	Eu/Tb
a	0.008	0.003	0.001	0.004	3:1
b	0.006	0.002	0.001	0.003	2:1
c	0.004	0.001	0.001	0.002	1:1
d	0.006	0.001	0.002	0.003	1:2
e	0.008	0.001	0.003	0.004	1:3
